# Photocatalytic decomposition of textile dyestuffs by photosensitive metal oxide catalysts

**DOI:** 10.3906/kim-2104-30

**Published:** 2021-10-19

**Authors:** Esra Yeliz ALTUN, Z. Tuba ŞİŞMANOĞLU, Gülin Selda POZAN SOYLU

**Affiliations:** 1 Department of Chemical Engineering, Engineering Faculty, İstanbul University-Cerrahpaşa, İstanbul Turkey; 2 Department of Chemistry, Engineering Faculty, Istanbul University-Cerrahpasa, Istanbul Turkey

**Keywords:** Degradation, textile azo dyes, photosensitive, metal oxide, CTAB

## Abstract

Textile azo dyes are one of the pollutants in waste water that adversely affect human and environmental health. Removal of these chemicals from wastewater is important for eco-system and human health. In this study, Bi_2_O_3 _nanoflakes and ZnO were synthesized by the co-precipitation method. Adsorption and photocatalytic degradation reactions were carried out to remove dyes (Victoria blue (VB) and Malachite green (MG)) from wastewater with the photocatalysts. In order to improve the activity of catalysts, cetyltrimethylammoniumbromide (CTAB) was added as a surfactant to pure oxide structures, and Bi_2_O_3_-CTAB and ZnO-CTAB catalysts were prepared. The structural and morphological properties of these catalysts were determined by BET, XRD, DRS, FTIR, and SEM analysis. It was found that the activity of the catalyst was improved by adding surfactant to the Bi_2_O_3_. The total mineralization of VB dye was completed in 60 min under sunlight with Bi_2_O_3_-CTAB catalyst. However, the degradation of the MG dye with the same catalyst under UV-C irradiation could be completed in 120 min.

## 1. Introduction 

Organic substances in wastewater are an important environmental problem. These pollutants adversely affect the ecosystem and human health [1]. Organic pollutants must be removed from wastewater before they are released into the natural environment. Paint is an important environmental pollutant affecting the eco-environment. The presence of dyes in wastewater reduces light transmission in the water environment and adversely affects photosynthetic activity. It also causes the formation of toxic and carcinogenic products [2].

Textile dyes have an important place among chemical pollutants and once mixed with water. They are difficult to remove from water due to their synthetic origin and complex molecular structure. It is thought that approximately one hundred thousand commercial paints are produced between 7.10^5^–10^6^ tons per year [3].

Some physical and chemical methods are widely used in the removal of dyes from wastewater [4–6]. These methods show some disadvantages in removing azo dyes. The most important of these is that the waste problem occurs during the treatment of dyes from aqueous solutions [7].

In this context, in biological treatment processes where no secondary waste problem is experienced, the treatment level is very low and insufficient due to the bio-carcinogenic and toxic nature of the dyestuffs [8]. Treatment of waste water containing dyes in recent years focused on advanced oxidation processes (IOP) [9]. The heterogeneous photocatalytic oxidation method included in the IOP enables the transformation of pollutants into harmless and/or less harmful components by degradation of pollutants in the presence of UV light and semiconductor metal oxides (ZnO, TiO_2_, CeO_2_) [10–12]. Recently there has been wider attention towards undoped and single oxide semiconductors which are nontoxic, stable, visible light active, and equally efficient photocatalysts. Among these materials, Bi_2_O_3_ and ZnO are the best candidates as photocatalytic materials because of their nontoxic nature, simple, and easy routes of synthesis, suitable band gap, and high photoluminescent properties. Additionally, surfactants have been often used for increase the photodegradation efﬁciency in UV–TiO_2_ systems [13–14]. In this study, photodegradation of dyes with Bi_2_O_3_-CTAB and ZnO-CTAB catalysts was examined. The effects of surfactant additive and different light sources on the removal of hazardous dyes from wastewater were investigated. Moreover, the relationships between the catalyst morphologies and the photocatalytic activities were also investigated by using varied characterization methods such as scanning electron microscope (SEM), diffuse reflectance spectroscopy (DRS), Brunauer, Emmet and Teller (BET), fourier transform infrared (FTIR), and X-ray diffraction (XRD).

## 2. Experimental and methods

### 2.1. Materials

In this experimental study, essential materials were supplied commercially and utilized without additional purification process. These materials are bismuth (III) nitrate pentahydrate (98%; Alfa Aesar Company), zinc nitrate hexahydrate, cetyltrimethyl-ammonium bromide, CTAB, Victoria blue (VB), and Malachite green (MG) (>= 99%, Sigma Aldrich). 

### 2.2. Catalyst synthesis methods

Bi_2_O_3 _nanopowder was prepared by co-precipitation method. Bismuth nitrate pentahydrate (0.97 g) was dissolved in nitric acid-water solution. Sodium hydroxide solution (0.8 g) was prepared with 100 mL of distilled water. The prepared solutions were kept in ultrasonic water bath for 15 min. Sodium hydroxide solution was slowly added dropwise into the bismuth nitrate pentahydrate solution until pH 11 under continuous stirring. After adjusting the pH of the solution, it was heated in a magnetic stirrer until the temperature reached 75 °C. The solution reaching the desired temperature was stirred under continuous stirring for 2 h without changing the temperature. Bi_2_O_3_ particles formed as precipitate were filtered and washed first with distilled water, then with absolute ethanol. It was dried in the oven at 80 °C for 2 h. The particles obtained were calcined in the calcination oven at a temperature of 450 °C for 2 h by heating at 10 °C/min. Particles formed after calcination were ground and Bi_2_O_3_ catalyst was obtained.

ZnO nanopowder was prepared by co-precipitation method. Zinc nitrate hexahydrate (8.93 g) was dissolved in 50 mL of distilled water and kept in an ultrasonic water bath at 50 °C for 10 min. The prepared solution was heated to 65 °C in a magnetic stirrer. When the temperature was stabilized, it was adjusted to pH = 10 with ammonia solution (25%). The solution, the pH of which was adjusted, was left to stir for 2 h at 65 °C in a magnetic stirrer. Precipitated ZnO particles were filtered and washed with distilled water for 2 h It was dried in an oven at 100 °C for 20 h. The particles obtained were calcined at 500 °C for 5 h by heating at 10 °C/min in the calcination oven. The calcined product was ground in the ball mill for approximately 10 min.

Preparation of surfactant loaded catalysts was used cetyltrimethyl-ammonium bromide (CTAB) as surfactant. CTAB was added over Bi_2_O_3_ and ZnO by the solid state dispersion method (SSD). This method is based on physical mixing of solid materials.

In the solid state dispersion method, catalysts (Bi_2_O_3_, ZnO) and the surfactant (CTAB) were weighed 1: 1 by weight and put into the ceramic mortar. They were thoroughly crushed with the help of ceramic drumsticks and mixed together. This process was continued until the particles of both substances became indistinguishable from each other. The mixing process was continued by adding a few drops of absolute ethanol to the desired substances with the help of dropper. A few more drops of absolute ethanol were added to the mixture, which reached the consistency of cake, and put into the oven to dry. The drying process was carried out for 90 min at 110 °C.

The particles obtained at the end of the drying process were calcined at 150 °C for 5 h by heating at 10 °C/min in the calcination oven. The calcination temperature was determined to be suitable for the decomposition temperature of CTAB. The calcined product was ground in the ball mill for approximately 10 min.

### 2.3. Catalyst characterization 

Total catalyst surface area of the catalyst was measured by nitrogen adsorption/desorption using a Quantachrome instrument. All catalysts were degassed under vacuum at 200 °C for 4 h. Crystallographic structure of catalyst were determined by X-ray powder diffraction using CuKα radiation (λ = 1.54056 Ε) with a Rigaku D/Max-2200 powder x-ray diffractometer. Before the analysis was run, the samples were gently granulated in an agate mortar to reduce the required orientation. Patterns were recorded at scan speed 2 degree of 2θ in the range of 10–90 °. The average crystallite size (D_avg_) was computed using the Debye–Scherrer equation. The powders were examined with a high resolution scanning electron microscope (SEM) (JEOL/JSM-a6335F) for possible differences in morphologies and size distributions of the powders. 

FT-IR spectra were examined by FT-IR spectroscopy (Perkin Elmer Precisely Spectrum One). KBr powder was used to prepare KBr pellets for samples. The samples were acquired as 100 scans with 4 cm^−1 ^resolutions. Optical energy gap of nanopowders were carried out by a double–beam UV-Shimadzu 3600 UV-Vis-NIR spectrophotometer equipped a diffuse reflectance (DR) accessory.

The energy of band gap for the catalyst was evaluated by using the Kubelka–Munk formula with Tauc’s relation (Eq. (1)), which derived from DRS measurement.

(1)(hnF(Ra))1/n=A(hn-Eg)

In this expression, hν is the energy of a single proton, α is the optical absorption co-efficiency, and *E*_g _is the optical band gap energy, A is constant for direct band gap transitions, the value of exponent parameter n denotes the nature of sample transition (n = ½ is used for the catalyst). R is the absolute reflectance value; F(R) is proportional to the absorption coefficient (α).

### 2.4. Studies on photocatalytic activity 

In this study, photocatalytic degradation reactions of azo dyes (Victoria blue, Malachite green) were carried out in a single neck reactor made of quartz material with a volume of 100 mL. The reactions were carried out under UV-B, UV-C, visible light, and natural sunlight. The reactions were carried out carried out under natural sunlight The photoreactor was placed on the magnetic stirrer and continuous mixing was performed. We compared the photcatalytic activities for different light source (UV-B, UV-C, and visible lamp) in a photoreactor system. 

After 100 mg of catalyst was added to the photoreactor, 50 mL of 25 ppm aqueous dye solution was slowly added and the mixture was kept in an ultrasonic bath for 2–3 min. All reactions were subjected to mixing without light (in the dark) for 30 min. At the end of the dark phase, samples were taken by giving light. This process was continued for 2 h. Samples were taken into centrifuge tubes at certain time intervals and centrifuged to separate the solid/liquid phase from each other. After centrifugation, the clear liquid remaining in the upper part of the tubes was taken to be analyzed in the UV-vis device. 

### 2.5. Adsorption process

The experimental studies were carried out on the removal of azo dyes (victoria blue and malachite green) using all the synthesized catalysts (Bi_2_O_3_, ZnO, CTAB-Bi_2_O_3_, and CTAB–ZnO). Adsorption studies were tested for varied temperatures (25 °C, 40 °C, 55 °C) and different initial concentrations (25 ppm, 50 ppm, 75 ppm, 100 ppm) for isotherm studies.

The stock solution for the each of dyes was prepared as 250 ppm. The 0.01 g catalyst samples (Bi_2_O_3_ and Bi_2_O_3_-CTAB) were interacted with 10 mL of VB solutions for 60 min at 25, 40, 55 °C and aliquots were taken every 15 min for spectrophotometric analysis. The same procedure was performed for the MG solution. However, the adsorption isotherm times of MG dye on ZnO and ZnO-CTAB catalysts were determined as 90 min. Adsorption studies were performed in brown glass bottles in a shaker water bath at 250 rpm. During the adsorption process, samples were taken at certain time intervals and put into centrifuge tubes. It was centrifuged to separate the solid/liquid phase formed. The clear liquid remaining on the upper part of the tubes was taken and the absorbance values ​​were measured in the UV-vis device. The concentrations corresponding to the measured absorbance values ​​were calculated using the standard graphs. In addition, the adsorption isotherms were tested by using Langmuir and Freundlich and Dubinin–Radushkevich (D-R) models.

### 2.6. Isotherms of adsorption

**Figure 1:**
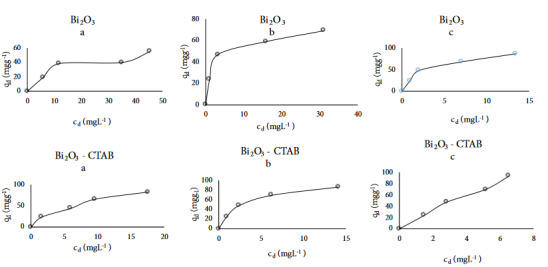
Adsorption isotherm curves of Victoria blue dye in the presence of Bi2O3 and Bi2O3-CTAB catalysts at different temperature a) 25 °C, b) 40 °C, and c) 55 °C.

Freundlich, Langmuir and Dubinin–Radushkevich (D-R) isotherm methods were applied for the results of adsorption of VB and MG dyes. The adsorption capacity of the sorbent (q_d _mg g^–1^) was calculated [15]. Adsorption isotherm type graphics of VB were drawn q_d_vs c_d_ at different temperatures. The Giles isotherm lines of Bi_2_O_3_ appear to be the same as L3, L2, and L2 types at 25, 40, and 55 °C respectively as seen in Figure 1. However, Giles isotherm types of Bi_2_O_3_-CTAB were found L4, L2, and L3 types at 25, 40, and 55 °C. respectively as shown in Figure 1. 

**Figure 2:**
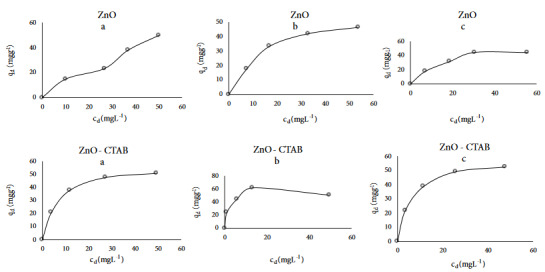
Adsorption isotherm curves of Malachite green dye in the presence of ZnO and ZnO-CTAB catalysts at different temperature a) 25 °C, b) 40 °C, and c) 55 °C.

Adsorption isotherm type graphics of MG were drawn q_d_vs c_d_ at different temperatures. While the Giles isotherm lines of ZnO are appearing L4 both 25 °C and 55 °C this line is the same as L2 for 40 °C as seen in Figure 2. However, Giles isotherm types of Bi_2_O_3_/CTAB were found L2, L5, and L2 types at 25, 40 and 55 °C, respectively as shown in Figure 2 [16]. 

These L type explain that the first monolayer line occurs and then multilayer line during adsorption process. L curves were described by Langmuir isotherms. The first curve is indicated molecules adsorbed plain on the surface, the second curve is shown in the perpendicular oriented adsorbed ions with particularly strong intermolecular attraction. These cases suggest that the adsorbed ions may imply united into very large stacks before adsorption happen. The L curve occurs in probably the plurality of conditions of adsorption at dilute solution.

Langmuir isotherm: The Langmuir equation is represented as follows (2): 

(2)1qd=1qmax.bcd+1qmax

Here, q_max_ (mg g^–1^) and b (L mg^–1^) expressions have the same meaning as those used in Equation 2 in the study of Duran et al. [13] 1/q_d_ versus 1/c_d_ with slope b and intercept 1/q_max_ is obtained (Tables 1–2). 

**Table 1:** The crystallite size, specific surface area, band gap, and morphology of materials.

Catalyst	Crystallite size	SBET	Band gap	Morphology
	nm	(m2g–1)	(nm)	
ZnO	65	28	2.93	Hexagonal
Bi2O3	42	15	2.83	Monoclinic
ZnO-CTAB	33	8	2.87	Hexagonal
Bi2O3-CTAB	23	9	2.59	Monoclinic

Freundlich isotherm: The Freundlich isotherm model was applied by the following 

(3)qd=KFcdn

The linear equation of the Freundlich isotherm was fitted as given below.

logq_d_ = log K_F _+ n log c_d_, (4)

where q_d_ is the amount adsorbed (mg g^–1^). C_d _the equilibrium concentration in aqueous phase (mg L^–1^). K_F_ and n are adsorption capacity and adsorption intensity, respectively.

The D-R isotherm is an experimental adsorption model applied with Gaussian energy distribution to express the adsorption mechanism to heterogeneous surfaces. This isotherm was specifically designed to separate the physical and chemical adsorption of adsorbate ions.

The D-R isotherm is stated as follows:

lnX_e_ = lnX_m_-K_DR _ε^2 ^(5) 

ε = RT ln(1+1/C_e_), (6)

where Ce (mg L^–1^) is dyes concentration at equilibrium, e is Polanyi potential, X_m_ (mg g^–1^) is the highest capacity of the adsorbent, the constant K_DR_ is concerned to the mean free energy of adsorption per mole of adsorbate. This energy is calculated the following equation (7);

(7)E=12KDR

Adsorption energy (E) value gives information about the mechanism of adsorption. 8 < E > 16 is kJ.mol^–1 ^the mechanism is ion exchange. E < 8 kJ.mol^–1^ is physical adsorption and E > 16 kJ.mol^–1^ is chemical adsorption.

## 3. Result and discussion

### 3.1. Characterization results

The surface areas of the samples were determined through adsorption of inert gas such as N_2 _for the purpose of observing the impact of surfactant addition on physical properties of the catalysts. The results were listed as given in Table 1. The surface area of pure Bi_2_O_3_ and ZnO are 15 and 28 m^2^/g, respectively. With the addition of surfactant, the surface areas of ZnO and Bi_2_O_3 _catalysts decreased. The reason for this change in surface area is thought to be related to the structure and size of the CTAB molecule participating in pure oxide structures.

**Figure 3:**
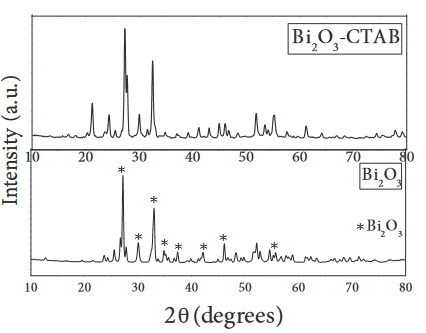
XRD patterns of Bi2O3 and Bi2O3-CTAB catalysts.

**Figure 4:**
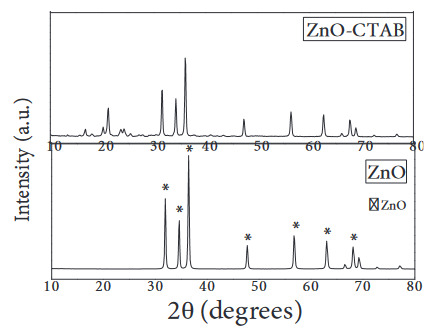
XRD patterns of ZnO and ZnO-CTAB catalysts.

XRD diagrams of the catalysts are given in Figures 3–4. It was understood that there was no change in the structure of the catalyst with the addition of surfactant, and the phases formed were compatible with the crystal structure of monoclinic Bi_2_O_3_ (JCPDS 41-1449) and hexagonal ZnO (JCPDS 36-1451).

**Figure 5:**
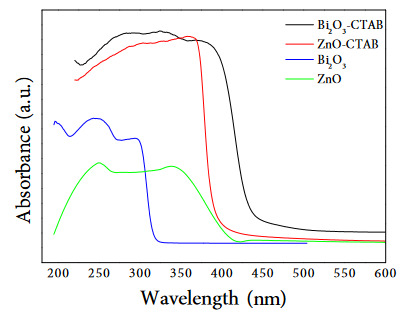
DRS spectra of Bi2O3, ZnO, Bi2O3-CTAB and ZnO-CTAB catalysts.

The band gap energies of the oxide structures were determined by diffuse reflectance spectroscopy (DRS) and the results are shown in Figure 5. According the DRS results, it was seen that there was a red shift, that is, a shift to higher wavelengths by adding CTAB to Bi_2_O_3_ and ZnO catalysts. It is noteworthy that this shift in wavelength is more in the Bi_2_O_3_ structure. This result indicates that there is a shift towards the visible region. Bi_2_O_3_-CTAB has a lower transmission band that increases its absorption ability in sunlight. The band gap energy values decreased with the addition of the CTAB addition to Bi_2_O_3_ and ZnO structure.

**Figure 6:**
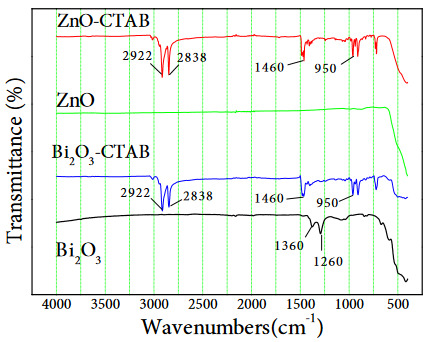
FTIR spectra of Bi2O3, ZnO, Bi2O3-CTAB and ZnO-CTAB catalysts.

FTIR-ATR analysis was carried out in order to prove the bonds and stresses in the structure of the prepared catalysts, to determine the existing functional groups and to determine the hydroxyl group that is important in photocatalytic reactions. The ATR spectra obtained as a function of wavelength are shown in Figure 6.

In the pure Bi_2_O_3_ catalyst, the Bi-O-Bi vibration peak around 1260 cm^−1^ and a C-H peak around 1360 cm^−1^ were seen. It was observed that these two peaks disappeared and some new peaks were formed with the addition of CTAB to the structure. The stress ν (C–N) at peak values of 912–962 cm^−1^ indicates the CH_2_ group belonging to the CTAB structure at 1460 cm^−1^, and the CH_3_ structure at 2838 and 2922 cm^−1^ values. The occurrence of these stresses in the CTAB added catalyst indicates that the added surfactant does not degrade after calcination and is added to the Bi_2_O_3_ structure. In FTIR-ATR analysis, similar peaks were observed after CTAB was added to the pure ZnO catalyst.

**Figure 7:**
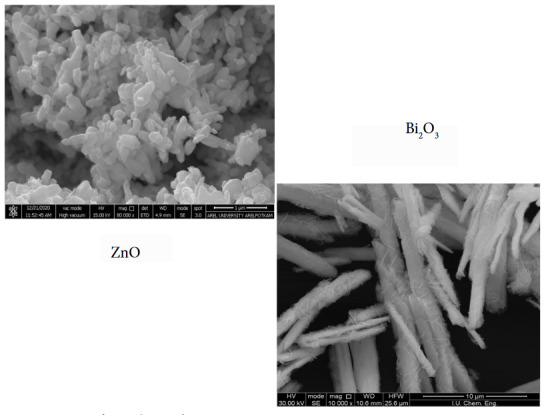
SEM images of Bi2O3and ZnO catalysts.

The surface morphology of the ZnO catalyst was illuminated in detail by SEM analysis. SEM images of pure ZnO and Bi_2_O_3_ are given in Figure 7. The clusters in cubic form were observed in the pure ZnO structure. However, rod-like structures of different lengths and thicknesses draw attention in the SEM image of Bi_2_O_3_ structure.

**Figure 8:**
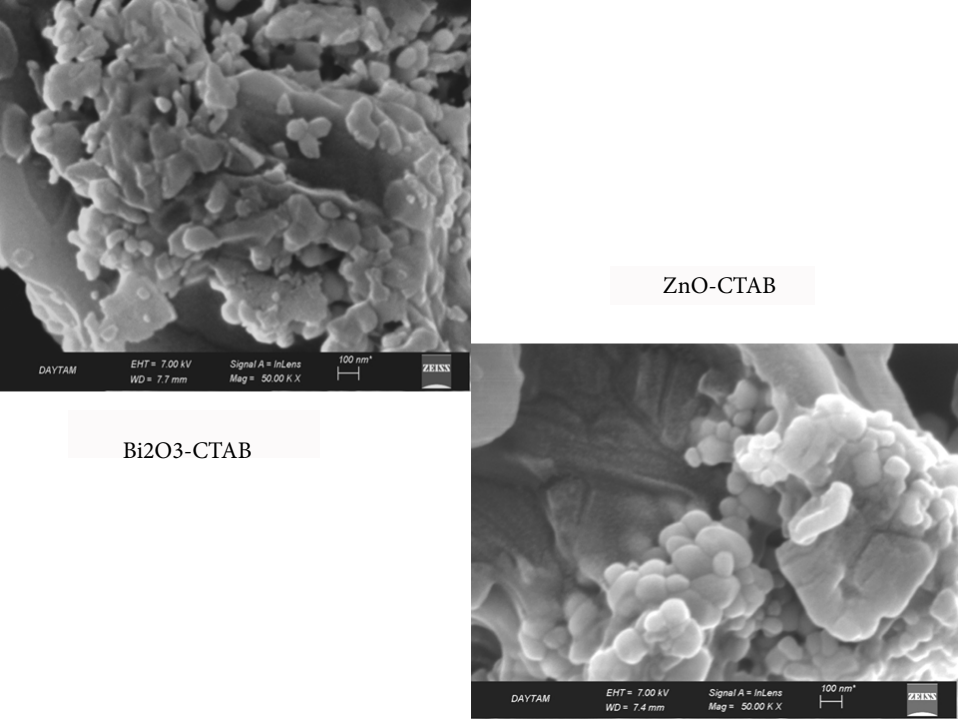
SEM images of Bi2O3-CTAB and ZnO-CTAB catalysts.

After addition of surfactant (Figure 8), it is understood that the spherical structure of the pure ZnO catalyst has not changed, but has an inhomogeneous particle distribution. In addition, it is observed that large and small mixed grains are together and the grain size is reduced. This change had a positive effect on photocatalytic activity. This situation confirms that CTAB has entered the structure as mentioned in the FTIR analysis. As seen in the XRD results, surfactants have a structure that reduces the particle size and thus affects the particle morphology.

Unlike the ZnO-CTAB structure, it was observed that by adding surfactant to the Bi_2_O_3 _structure, the rod-like structures came together and formed a pile (Figure 8). In addition, the agglomeration of small particles and a small number of rod-like structures draw attention. The reduction of the grain structure and the surface appearance in the form of layers is due to the aggregation tendency of the surfactant adsorbed on the crystal surface. Van der Waals interaction between molecules caused agglomeration. As a result, with the addition of CTAB, the Bi_2_O_3_ surface has changed considerably and smaller structures have been formed. This change had a positive effect on photocatalytic activity.

### 3.2. The results of adsorption studies

Adsorption intensities of VB on Bi_2_O_3_ and Bi_2_O_3_/CTAB at each of temperatures were found n < 1 as seen in Table 2. This result shows that the binding energy decreased with increasing the surface concentration and the adsorption was occurred. It seems that the adsorption capacities (K_F_) increased with increasing temperatures for Bi_2_O_3_ catalyst. However, the adsorption capacities of Bi_2_O_3_-CTAB catalyst did not changed with increasing temperatures. For Freundlich isotherm correlation coefficients average of adsorption of VB on Bi_2_O_3_ and Bi_2_O_3_-CTAB are 88%, 98% respectively. Table 1 appears that the maximum amount of substance adsorbed in the single layer (q_max_) is 50 ^o^C > 40 ^o ^C >25 ^o^C for the Langmuir and D-R isotherms. The best correlation coefficients average of adsorption of VB on Bi_2_O_3_ and Bi_2_O_3_/CTAB were found for Langmuir isotherm as 97% and 99% respectively. According to Langmuir model, the highest values of q_max_ from adsorbed VB on 0.01 g Bi_2_O_3_ and Bi_2_O_3_/CTAB were found to be at 50 °C temperature as 111 mg g^–1^, 625 mg g^–1^, respectively as seen in Table 2. According to D-R adsorption isotherm, the adsorption energy of VB on Bi_2_O_3_ and Bi_2_O_3_/CTAB were found to be E < 8 kJ.mol^–1^ for each of temperatures. This result indicates that the adsorption mechanism is described to be physical adsorption.

**Table 2:** Adsorption isotherm parameters of Victoria blue dye.

25 °C	Langmiur Isotherm			Freundlich Isotherm			D-R Isotherm			
	qmax (mg.g–1)	b (L.mg–1)	R2	n	Kf (L.mg–1)	R2	qmax (mg.g–1)	Kdr (L.mg–1)	E (kJ.mol–1)	R2
Bi2O3	69	0.07	0.92	0.41	10.8	0.81	48	5.96	0.43	0.91
Bi2O3-CTAB	94	0.2	0.98	0.54	17.8	0.99	66	0.75	1.21	0.84
40 °C	Langmiur Isotherm			Freundlich Isotherm			D-R Isotherm			
	qmax (mg.g–1)	b (L.mg–1)	R2	n	Kf (L.mg–1)	R2	qmax (mg.g–1)	Kdr (L.mg–1)	E (kJ.mol–1)	R2
Bi2O3	73	0.47	0.99	0.29	27	0.90	61	0.35	1.76	0.96
Bi2O3-CTAB	105	0.40	1	0.46	28	0.96	74	0.34	1.79	0.93
55 °C	LangmiurIsotherm			Freundlich Isotherm			D-R Isotherm			
	qmax (mg.g–1)	b (L.mg–1)	R2	n	Kf (L.mg–1)	R2	qmax (mg.g–1)	Kdr (L.mg–1)	E (kJ.mol–1)	R2
Bi2O3	111	0,30	0.98	0.45	28.5	0.93	76	0.37	1.72	0.96
Bi2O3-CTAB	625	0,027	0.98	0.89	17.3	0.99	88	0,83	1.15	0.94

Adsorption intensities of MG on ZnO and ZnO-CTAB at each of temperatures were found n < 1 as seen in Table 3. It seems that the adsorption capacities (K_F_) varied irregular with increasing temperatures for ZnO and ZnO/CTAB catalysts. While the maximum amount of substance adsorbed in the single layer (q_max_) is changing 25 °C > 40 °C > 55 °C for the Langmuir, but this value changed (X_m_) 50 °C > 40 °C >25 °C for D-R isotherms. Both Freundlich and Langmuir correlation coefficient average of adsorption of MG on ZnO and ZnO/CTAB were found to be 87% and 98%, respectively. The best correlation coefficients average of adsorption of MG on ZnO and ZnO-CTAB found for Langmuir isotherm. According to Langmuir model, the highest values at monolayer coverage (q_max_) of adsorbed MG on 0.01 g ZnO and ZnO-CTAB were found to be at 25 °C as 73 mg g^−1^ and 60 mg g^–1^, respectively as seen in Table 3. According to D-R adsorption isotherm, the adsorption energy of MG on ZnO and ZnO-CTAB were found to be E < 8 kJ.mol^–1^ at different temperatures. It means that the adsorption mechanism is found to be physical adsorption as shown in Table 3.

**Table 3:** Adsorption isotherm parameters of Malachite green dye.

t/ 5 oC	Langmiur Isotherm			Freundlich Isotherm			D-R Isotherm			
	qmax (mg.g–1)	b (L.mg–1)	R2	n	Kf (L.mg–1)	R2	Xm (mg.g–1)	Kdr (L.mg–1)	E (kJ.mol–1)	R2
ZnO	73	0.025	0.95	0.74	2.5	0.93	39	18	0.28	0.75
ZnO-CTAB	60	0.134	1	0.35	14	0.93	47	2.66	0.72	0.96
t / 40 oC	Langmiur Isotherm			Freundlich Isotherm			D-R Isotherm			
	qmax (mg.g–1)	b (L.mg–1)	R2	n	Kf (L.mg–1)	R2	Xm (mg.g–1)	Kdr (L.mg–1)	E (kJ.mol–1)	R2
ZnO	70	0.046	0.99	0.49	7.5	0.93	44	9.6	0.38	0.98
ZnO-CTAB	59	0.67	0.96	0.21	27	0.70	53	0.29	2.17	0.91
t/55 oC	Langmiur Isotherm			Freundlich Isotherm			D-R Isotherm			
	qmax (mg.g–1)	b (L.mg–1)	R2	n	Kf (L.mg–1)	R2	Xm (mg.g–1)	Kdr (L.mg–1)	E (kJ.mol–1)	R2
ZnO	64	0.054	0.99	0.47	7.2	0.91	43	8.5	0.40	0.93
ZnO-CTAB	58	0.17	0.99	0.34	15.2	0.95	58	1.96	0.84	0.94

### 3.3. Photocatalytic activity results

In photocatalytic reactions, before the light is turned on, the catalyst and the dye to be decomposed are stirred for 30 min without light in order to ensure adsorption. The dark phase period continues until the adsorption equilibrium is reached [17]. In this study, the activities of the surfactant added catalysts were tested in the photocatalytic degradation reactions of the dyestuffs. We based our experiments on the Langmuir–Hinshelwood (L-H) kinetic model [18]. It can be expressed by Equation 8 below: 

ln(C_o_/C) = k_obs_t. (8)

Assuming that *C = C* *_o_* at t = 0 with low initial BPA concentration, where t is the given irradiation time and *k* *_obs_* is the rate constant of the observed pseudo-first order reaction. After experiments, we calculated the photocatalytic degradation efficiencies and reaction rates for the different photocatalysts and the numerical values of gain are presented in Table 1.

The degradation efficiency (R%) of BPA was calculated by Equation (9):

(9)R%=C0-CC0x100

Within the scope of this study, in the presence of ZnO, Bi_2_O_3_, ZnO-CTAB, and Bi_2_O_3_-CTAB catalysts, which were prepared and characterized, UV-B, UV-C, Victoria blue (VB), and Malachite green (MG) textile dyes under visible light and sunlight degradation reactions were carried out.

**Figure 9:**
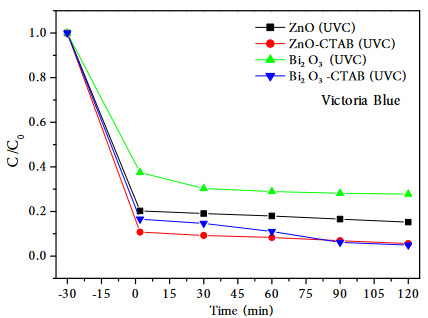
The photocatalytic degradation of Victoria Blue in Bi2O3, Bi2O3-CTAB, ZnO, ZnO-CTAB catalysts.

**Figure 10:**
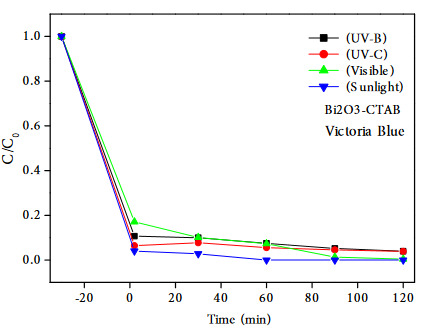
The photocatalytic degradation of Victoria blue on Bi2O3-CTAB catalyst with different irradiation source.

Figure 9 shows the photocatalytic degradation of Victoria blue in the different catalysts under UV-C illumination at different irradiation time. When the color removals of VB dyestuff at the end of 120 min, the photocatalytic efficiencies are sorted in descending order: Bi_2_O_3_-CTAB (95.04%) > ZnO-CTAB (94.36%) > ZnO (84.76%) > Bi_2_O_3_ (72.20%). Since the highest activity was achieved in the presence of Bi_2_O_3_-CTAB catalyst, reactions were also carried out under visible light and sunlight. The photodegradation results of VB on Bi_2_O_3_-CTAB catalyst with different irradiation source are shown in Figure 10. As a result of these reactions, 99.68% color removal was achieved in 120 min under visible light, and complete degradation (100% color removal) within 60 min under sunlight. 

**Figure 11:**
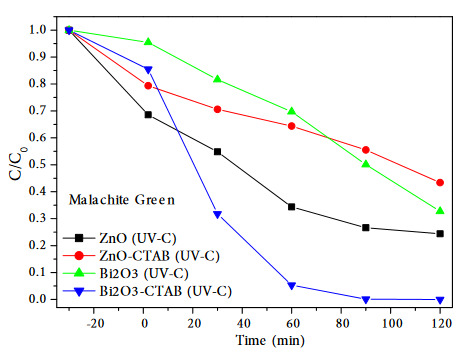
The photocatalytic degradation of Malachite green in Bi2O3, Bi2O3-CTAB, ZnO, ZnO-CTAB catalysts.

Pare et al. studied the degradation of Victoria blue dye B in aqueous suspension of zinc oxide. In the study, they realized that the total mineralization of VBB (9.0 x 10^−4^ moldm^−3^) was completed in 8 h of irradiation at the optimum reaction conditions [19]. The color removals of MG dyestuff were performed under UV-C radiation in the presence of prepared catalysts. The efficiencies were calculated as Bi_2_O_3_-CTAB (100%)>ZnO (97.27%)> Bi_2_O_3_ (67.26%)>ZnO-CTAB (64.37%). As a result in Figure 11, the complete decomposition was achieved within 120 min under UV-C irradiation with Bi_2_O_3_-CTAB catalyst.

Chen et al. prepared Bi_2_WO_6_ nanostructure and investigated photocatalytic degradation of MG dye. The activity results showed the degradation efficiency with Bi_2_WO_6 _photocatalyst in the dark for 90 min is only 13.6%.

## 4. Conclusion

The effect of surfactant additive with Bi_2_O_3 _and ZnO nanoparticles on the photocatalytic degradation of dyes (VB, MG) were investigated. The photocatalytic activities of catalysts were compared for different light sources. Considering the prepared catalysts and all the reactions, it is understood that sunlight provides more effective degradation than UV light sources.

It was seen that the addition of CTAB to the pure Bi_2_O_3_ catalyst enhanced the photocatalytic activity and increased color removal. However, it has been observed that CTAB addition to ZnO catalyst does not have an activity improving effect. With the addition of CTAB surfactant on Bi_2_O_3_, the degradation efficiency of VB under sunlight was improved, complete degradation was achieved within 60 min. In the presence of the surfactant added Bi_2_O_3_-CTAB catalyst, the Malachite green was decomposed 100% under UV-C irradiation within 120 min.

Different adsorption isotherms were examined. It was seen that the adsorption mechanism was physical adsorption for all adsorption trials according to the D-R isotherm. According to Freundlich isotherm data, for all adsorption experiments, the bonding energy decreased with increasing surface concentration. This showed that adsorption is a preferred adsorption. 

The adsorption of VB and MG dyes on the catalysts was observed in which correlation coefficient best suited the Langmuir model. The adsorption of VB on Bi_2_O_3_-CTAB was the highest. The maximum adsorption capacity (q_max_) was found to be 625 mg g^−1^ at 50 °C temperature. The maximum adsorption capacity of MG on ZnO was found to be 73 mg g^−1^ at 25 °C temperature. The results of adsorption energy under these working conditions showed that the whole adsorption process is physical adsorption.
